# Assessment of Hypervirulent *Klebsiella pneumoniae* Isolates Using Multilocus Sequence Typing in West of Iran

**DOI:** 10.1002/mbo3.70049

**Published:** 2025-08-19

**Authors:** Mohammad Hossein Haddadi, Nourkhoda Sadeghifard, Saeed Khoshnood, Abbas Maleki, Sobhan Ghafourian, Hassan Valadbeigi

**Affiliations:** ^1^ Clinical Microbiology Research Center Ilam University of Medical Sciences Ilam Iran; ^2^ Department of Microbiology, School of Medicine Ilam University of Medical Sciences Ilam Iran

**Keywords:** hypervirulent, *K. pneumonia*, MLST, sequence type

## Abstract

**Background:**

Hypervirulent *Klebsiella pneumonia* (hvKP) has been considered due to severe infections such as pyogenic liver abscess, pneumonia, meningitis, endophthalmitis and necrotizing fasciitis. Due to the importance of the subject, hv*KP* isolates were investigated in the current study.

**Methods:**

A total of 52 non‐repetitive *Klebsiella pneumonia (K. pneumonia*) isolates were collected from clinical specimens (pleural fluid, blood, urine and trachal aspiration) of hospitalized patients in three well‐known hospitals in Ilam, west of Iran. Phenotypic tests, including the string test and potassium tellurite resistance, and molecular methods such as polymerase chain reaction (PCR) for *iucA*, *iroB*, *iutA*, and *rmpA* genes were used for hvKP diagnosis. In addition, multilocus sequence typing (MLST) was used to clarify the genetic relationships between isolates.

**Results:**

The *mrkD*, *iroB*, *iucA*, *iutA*, and *rmpA* genes were present in 6 (11.53%), 4 (7.7%), 12 (23.1%), 5 (9.6%), and 8 (15.4%) of the *K. pneumoniae* isolates, respectively. Eight isolates from different sources were identified with new sequence types (STs). These newly identified STs include ST‐7466, ST‐7467, ST‐7468, ST‐7469, ST‐7470, ST‐7471, ST‐7472 and ST‐7473.

**Conclusions:**

We reported the eight new STs based on the MLST method for the phylogenetics of hvKP isolates. Our data indicated that no correlation between the phylogenetics of hvKP isolates. So, more extensive studies are needed to better understand the epidemiology of hvKP.

## Graphical abstract

This study identified eight novel sequence types (STs) of hypervirulent *Klebsiella pneumoniae* using MLST in western Iran. The findings highlight the genetic diversity and public health threat posed by hvKP strains. Phenotypic and genotypic methods, including PCR and MLST, were used to identify hypervirulent *Klebsiella pneumoniae* isolates in western Iran, revealing eight novel sequence types and highlighting the genetic diversity of hvKP strains.

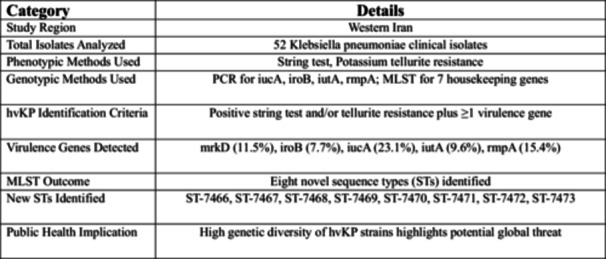

## 1 Introduction

*Klebsiella pneumonia* is recognized worldwide as an emerging pathogen (Chang et al. 2021). This increased attention can be attributed to the increase in severe *K. pneumoniae* infections, the emergence of antibiotic resistance and the obstacles to the development of effective therapeutic interventions (Navon‐Venezia et al. 2017; Wang et al. 2020). *K. pneumoniae* has emerged as a major pathogen causing hospital‐acquired infections, particularly in immunocompromised patients (Priyanka et al. 2020). It is now the second most common cause of Gram‐negative bacteremia with a mortality rate between 27.4% and 37.0%. The hypervirulent (hv)isolates of *K. pneumoniae* pose an even greater threat and lead to alarmingly high mortality rates (Sharon Dorothy 2020). The first documented case of liver abscess caused by *K. pneumoniae* was reported in Asia in the 1980s (Parrott et al. 2021). Since then, there has been a growing number of severe infections associated with hvKP, including meningitis, endophthalmitis and necrotizing fasciitis, particularly in China (Fan et al. 2022). This particular case has been termed hvKP because of its higher pathogenicity and higher mortality compared to classical *K. pneumonia* (cKP). Since its first identification, hvKP has been recognized as a distinct circulating pathotype of *K. pneumoniae*, characterized by its high virulence and ability to cause severe infections. Hence, *K. pneumoniae* can be divided into two different types, namely cKP and hvKP (Alharbi et al. 2023). hvKP is attributed to the possession of various virulence factors, such as capsule (*rmpA*: important regulator of extracellular polysaccharide synthesis, capsule that forms mucilaginous colonies), siderophores (*iutA*: gene responsible for encoding aerobactin siderophores), lipopolysaccharides (LPS), and fimbriae (Parrott et al. 2021). These virulence factors play a decisive role in enhancing the pathogenicity of the bacterium (Alharbi et al. 2023). Understanding the prevalence of these genes is crucial for identifying populations at high risk of severe infection. In this study, we will focus on detecting genes such as *iucA*, *iroB*, *iutA*, and *rmpA* to monitor the virulence factors of *K. pneumoniae*. This approach aims to provide insights into the distribution of virulence‐associated genes and their potential impact on public health.

The key distinction is found in the string test, a widely used method for identifying hvKP. This test detects a hypermucoviscous phenotype, indicated by a string test measurement of more than 5 mm (Li et al. 2020). The hypermucoviscous phenotype is characterized by the production of an unusually thick and sticky bacterial capsule, which can enhance the pathogen's ability to evade the immune system and resist antibiotics. The accuracy of the semi‐qualitative test for clinical hvKP isolates can range from 70% to 90%, depending on factors such as colony conditions and operator technique (Russo et al. 2021). Another phenotypic method to detect hvKP isolates can be potassium tellurite based on Mac‐Conkey medium used as a selective medium (Liu et al. 2021). Therefore, hvKP isolates are identified in this study based on tellurite resistance and detection of different genes.

On the other hand, molecular techniques commonly used to characterize *K. pneumoniae* isolates in epidemiological investigations include random amplified polymorphic DNA, pulsed‐field gel electrophoresis, and amplified fragment length polymorphism (Arabzadeh et al. 2022). While these methods are commonly used to study localized outbreaks, their reproducibility across laboratories can be challenging. Moreover, they do not provide highly informative and unambiguous data, which limits their applicability in a broader epidemiologic context (Riley 2018; Gona et al. 2020). Multilocus sequence typing (MLST) is a molecular technique used to characterize bacterial isolates based on the sequences of multiple housekeeping genes, allowing for the identification and comparison of different isolates within a species (Maiden 2006). The use of nucleotide sequences provides accurate and reproducible results, making it an ideal tool for the characterization of bacterial genomes. Moreover, the ability of MLST to generate unique and transferable data facilitates its integration into global databases, ensuring its accessibility and usability in different research laboratories. Therefore, in this study, we utilized phenotypic and genotypic approaches to identify hvKP and employed MLST to analyze the phylogenetic relationships of hvKP isolates from tertiary care hospitals in Ilam, located in west Iran.

## 2 Methods

### 2.1 Sample Collection

A total of 75 samples, 52 non‐repetitive *K. pneumoniae* isolates were obtained from clinical samples of inpatients at three tertiary care hospitals—Razi, Emam Khomini, and Mostafa—located in Ilam, west of Iran, during January 2023 to June 2024. The isolates were extracted from various sources including pleural fluid, blood, trachal aspiration and urine. This study was approved by the Research Ethics Committee of Ilam University of Medical Sciences (IR.medilam.rec.1403.090). All patients provided written informed consent. This study was in accordance with the Declaration of Helsinki.

### 2.2 Bacterial Isolation and Culture

The bacterial isolates were identified using conventional biochemical laboratory techniques. The isolates were cultured on MacConkey agar media. Various standard biochemical tests were performed to identify and isolate *K. pneumoniae*, such as lactose fermentation, urease activity, citrate utilization and indole production. Pure colonies were isolated and preserved at −80°C for subsequent analysis.

### 2.3 Hypermucoviscous Characterization

#### 2.3.1 String Test

The string test based on the hypermucoviscous phenotype has been used extensively as a reliable marker for the presence of hypervirulent isolates of *K. pneumonia* (Russo et al. 2018; Chang and Ong 2022), such that isolates exhibiting the hypermucoviscous phenotype, known as hvKP, have been identified on the basis of a positive result of the string test. Conversely, isolates that showed a negative result were classified as cKP. A positive result of the string test was defined as the formation of a mucoviscous string measuring at least 5 mm in length obtained by elongation of a colony grown overnight on an agar plate at 37°C with a bacteriological loop.

### 2.4 Resistance With Potassium Tellurite

In the current study, a potassium tellurite agar culture was used as a rapid test. MacConkey potassium tellurite medium (MCKT) was prepared by first following the instructions for the preparation of MacConkey medium powder. A 1% solution of potassium tellurite was then incorporated into the medium (Wu et al. 2022; Latha et al. 2024).

The *K. pneumoniae* isolates were cultured in Columbia blood medium at a temperature of 35°C for 24 h. A bacterial suspension was then prepared by selecting three colonies and adding them to 5 mL of sterilized saline. The resulting suspension reached a turbidity concentration of 0.5 McFarland, which corresponds to a bacterial density of 1 × 10^8^ CFU/mL. After thorough shaking and mixing, a 10 μL bacterial suspension was evenly applied to MacConkey medium supplemented with potassium tellurite. The samples were then incubated at 35°C for 24 h and the results analyzed. Positive colony growth was indicated by the presence of pink or black colonies on the MCKT medium (Wu et al. 2022; Latha et al. 2024).

### 2.5 Detection of Virulence Genes by PCR

Previous studies have shown that the detection of *iucA*, *iroB*, *iutA* and *rmpA* genes is highly accurate in the identification of hvKP (Russo et al. 2024). So, in current study, the *iucA*, *iroB*, *iutA*, and *rmpA* genes were amplified by PCR and the amplification results were subsequently analyzed by gel electrophoresis.

In this study, the differentiation between hvKP and cKP was achieved by evaluating the presence or absence of specific virulence genes of *iucA*, *iroB*, *iutA*, and *rmpA* (Neumann et al. 2023). Consequently, *K. pneumoniae* isolates positive for *iucA*, *iroB*, *iutA*, and *rmpA* were categorized as hvKP (*iucA*+*/iroB*+*/iutA*+*/rmpA*+), while isolates lacking these genes were categorized as cKP (*iucA−/iroB−/iutA−/rmpA*−) (Neumann et al. 2023; Compain et al. 2014).

### 2.6 Genotype Confirmation of hvKP

In the current study, all positive string test with tellurite‐resistant *K. pneumoniae* were tested for the presence of the *iucA*, *iroB*, *iutA*, and *rmpA* genes. The isolates with one or more the *iucA*, *iroB*, *iutA*, and *rmpA* genes were considered as hvKp (Neumann et al. 2023; Compain et al. 2014).

### 2.7 DNA Extraction and Amplification of Housekeeping Genes

Whole DNA was isolated by BACTOKIA kit (Kiagen fanavar‐Iran) according to the manufacturer's instructions. The PCR amplification technique was used to amplify seven housekeeping genes (*gapA*, *infB*, *mdh*, *pgi*, *phoE*, *rpoB*, and *tonB*), with the specific primer sequences listed in Table 1. PCR condition was a denaturation step at 94°C for 20 s, followed by annealing at specific temperatures for different genes (50°C for most genes, 60°C for *gapA*, and 45°C for tonB) for 30 s. The extension step was performed at 72°C for 30 s. This entire cycling process was repeated 35 times. Before the actual amplification, a 2‐min denaturation step at 94°C was performed to initiate the reaction. Finally, after completing the cycling steps, a final extension at 72°C for 5 min was conducted (Diancourt et al. 2005).

**Table 1:** Primers used in current study for MLST typing of hvKP.

Locus (Putative function of gene)	Primers sequence (5→ 3)	Product length	Reference
*rpoß*	F: GGCGAAATGGCWGAGAACCA	1075	https://bigsdb.pasteur.fr/klebsiella/primers-used/
(RNA polymerase–ß subunit)	R: GAGTCTTCGAAGTTGTAACC		
*gapA*	F: TGAAATATGACTCCACTCACGG	662	
(glyceraldehydes 3‐phosphate dehydrogenase A)	R: CTTCAGAAGCGGCTTTGATGGCTT		
*mdh*	F: CCCAACTCGCTTCAGGTTCAG	756	
(malate dehydrogenase)	R: CCGTTTTTCCCCAGCAGCAG		
*pgi*	F: GAGAAAAACCTGCCTGTACTGCTGGC	566	
(phosphoglucose isomerase)	R: CGCGCCACGCTTTATAGCGGTTAAT		
*pohE*	F: ACCTACCGCAACACCGACTTCTTCGG	602	
(phosphoporin protein E)	R: TGATCAGAACTGGTAGGTGAT		
*infB*	F: CTCGCTGCTGGACTATATTCG	462	
(translation initiation factor 2)	R: CGCTTTCAGCTCAAGAACTTC		
*tonB*	F: CTTTATACCTCGGTACATCAGGTT	539	
(preplasmic energy transducers)	R: ATTCGCCGGCTGRGCRGAGAG		

The PCR purification kit (Bioneer, Korea) was used to purify the PCR products according to the manufacturer's instructions. This important step ensured accurate and reliable DNA sequencing results as it effectively eliminated all nonintegrated nucleotides, primers and contaminants. After purification, the purified PCR products were forwarded to AvinStemGene for DNA sequencing analysis.

### 2.8 MLST Typing

MLST analysis was performed according to the methodology developed by Diancourt et al (Diancourt et al. 2005). The MLST was performed with seven specific housekeeping genes (*gapA*, *infB*, *mdh*, *pgi*, *phoE*, *rpoB*, *tonB*) by PCR using corresponding primer pairsThe whole genome sequencing data were analyzed using MLST software (version: 2.23.0) to identify genetic variations in the seven conserved housekeeping genes among 8 isolates of *K. pneumoniae*. By comparing the sequences of these genes across all isolates, the specific STs of the hvKp. isolates were determined.

Alleles and STs were assigned using the MLST database developed specifically for *K. pneumoniae* (www.pasteur.fr/mlst/Kpneumoniae.html).

### 2.9 Statistical Analysis

The statistical analyses of data were performed using SPSS software, version 16.0 and Chi‐square tests were used to compare the data associated with hvKp and cKp isolates. Finally, the *p*‐values < 0.05 was considered statistically significant. The phylogenetic data analysis was conducted by PHYLOViZ software (https://online.phyloviz.net) (Ribeiro‐Gonçalves et al. 2016).

## 3 Results

### 3.1 Bacterial Isolation and hvKP Charectrization

From a total of 75 samples, 52 *K. pneumoniae* isolates were collected over a period of 6 months. Thirty‐four non‐repetitive isolates were positive for the string test or tellurite resistant. The frequency of cKP and hvKP found in this study shown in Table 2.

**Table 2:** Frequency of cKP and HvKP found in this study.

Samples	Frequency	Percent	Valid percent	Cumulative percent
Blood	11	21.0	21.0	21.0
Pleural fluid	11	21.0	21.0	42.0
Tracheal aspiration	8	15.0	15.0	57.0
Urine	22	42.0	42.0	100.0
Total	52	100.0	100.0	

### 3.2 String Test and Resistance to Potassium Tellurite Results

Of the 52 *K. pneumoniae* isolates, 25 isolates (48.07%) had a positive string test, indicating the presence of hypermucoviscosity. And also, the results of this study showed that 24 (46.2%) of the 51 *K. pneumoniae* isolates grew on MCKT medium and were considered tellurite‐resistant isolates (Table 3). In total, 34 (66.6%) non‐repetitive isolates were positive for the string test or tellurite potassium resistant.

**Table 3:** Characterization of phenotypic and genotypic collected isolates in current study.

	Phenotypic results		PCR results				
ID	String test	Potassium tellurite	*mrkD*	*iroB*	*iucA*	*iutA*	*rmpA*
1	_	_	_	_	+	_	_
2	+	_	_	_	+	+	_
3	_	+	_	_	_	_	_
4	_	_	_	_	+	+	_
5	+	+	_	_	_	_	_
6	_	_	+	_	_	_	_
7	+	_	_	_	_	_	_
8	+	_	_	_	_	_	_
9	_	+	_	+	_	_	_
10	+	+	_	+	_	_	_
11	_	_	_	_	+	_	_
13	+	_	_	_	_	_	_
14	+	+	_	_	+	_	_
15	_	_	+	_	_	_	+
17	+	+	_	_	+	_	_
21	+	_	_	_	_	_	_
22	_	_	_	_	+	_	_
23	+	+	_	_	_	_	_
25	+	+	_	_	+	_	_
26	_	_	+	_	_	_	_
27	_	+	+	_	_	_	_
29	+	+	_	_	_	_	_
30	_	_	_	+	_	_	_
31	_	+	_	_	_	_	+
32	_	_	_	_	_	_	+
33	_	+	_	_	_	_	+
35	+	_	_	_	_	_	_
36	_	_	+	_	_	_	_
37	_	_	_	_	_	_	+
38	_	+	_	_	_	_	_
39	_	_	_	_	+	_	_
40	+	_	_	_	_	_	+
41	_	_	_	_	_	_	+
43	+	+	_	_	_	+	_
46	+	+	_	_	+	_	_
51	+	+	_	_	_	_	_
56	_	_	_	_	+	_	_
57	+	_	_	_	_	_	_
58	+	+	_	_	+	_	_
60	+	+	+	_	_	_	_
62	+	_	_	_	_	_	_
63	_	_	_	_	_	_	+
64	_	+	_	_	_	_	_
65	_	_	_	+	_	_	_
66	_	+	_	_	_	_	_
68	+	+	_	_	_	_	_
69	_	+	_	_	_	+	_
70	_	_	_	_	_	+	_
71	+	_	_	_	_	_	_
72	_	+	_	_	_	_	_
73	+	+	_	_	_	_	_
75	+	_	_	_	_	_	_
Total: 52	25	24	6	4	12	5	8

### 3.3 Present of Genes *iucA*, *iroB*, *iutA*, and *rmpA*

In the *K. pneumoniae* isolates, the genes *mrkD*, *iroB*, *iucA*, *iutA*, and *rmpA* were found in 6 (11.53%), 4 (7.7%), 12 (23.1%), 5 (9.6%), and 8 (15.4%) of the samples, respectively (Table 3).

### 3.4 Confirmation of hvKP With Phenotypic and Genotypic

In this study, string test, resistance to potassium tellurite and the simultaneous presence of one positive genes was referred to as hvKP (Table 4). The results of the study indicate that the majority of genes had at least positive results, with the exception of the *iucA* gene, which had four positive cases. Eight isolates were included in the current study because they were positive string test and potassium tellurite resistance with simultaneously positive with one positive genes in *k. pneumonia*.

**Table 4:** String teas, potassium tellurite and virulence‐associated genes detection of the isolates in current study.

ID	String test	Potassium tellurite	*mrkD*	*iroB*	*iucA*	*iutA*	*rmpA*
10	P	P	N	P	N	N	N
14	P	P	N	N	P	N	N
17	P	P	N	N	N	N	P
25	P	P	N	N	P	N	N
43	P	P	N	N	N	P	N
46	P	P	N	N	P	N	N
58	P	P	N	N	P	N	N
60	P	P	P	N	N	N	N
	SP		SP + *mrkD*	SP + *iroB*	SP + *iucA*	SP + *iutA*	SP + *rpmA*
	8		1	1	4	1	1
Total			8				

*Note:* String test + potassium tellurite: SP.

### 3.5 MLST

In this study, eight isolates were selected and subsequently sequenced.

### 3.6 Nearest SD in Current Study

The isolate with the number 10 id, was most closely related to the sequence types ST231, ST2947, ST5785, ST5970, ST6259, and ST6459. Similarly, isolate 14 showed the closest matches with ST26, ST39, ST163, ST175, ST224, ST238, ST253, ST260, ST323 and ST402. The remaining isolates with the closest STs can be found in Table 5.

**Table 5:** Allelic profiles in hvKP and nearest ST in this study.

	Alellic profile							
ID	*gapA*	*infB*	*mdh*	*pgi*	*phoE*	*rpoB*	*tonB*	Nearest ST
10	2	6	70	8	467	81	77	231,2947,5785,5970,6259,6459
14	2	1	469	322	9	1	161	26,39,163,175,224,238,253,260,323,402
17	3	77	3	322	9	4	42	2551,580,839,1527,2541,2590,2729,2844
25	3	4	435	393	7	48	940	3486
43	3	4	435	383	7	6	38	147,1137,1488,1561,1880,2084,2358, 486, 3491,3740,4012,4843,4870,5352,5616,5819,6471, 6873,7166
46	2	1	2	322	9	1	911	39,224,402,412,429,538,593,667, 813,947,2236,2451,2520,2800,3165,3423,3479,4255, 5942,6807,6859
58	2	85	435	431	26	4	77	211,231,322,411,474,807,2217,2439,3171,3321,3620,3652,5532,5785,5814,5970
60	2	6	38	1	16	7	77	310,4804,5828,6459,6933

### 3.7 New Assigned ST for hvKP

Eight isolates from different sources (urine, urinary catheter, blood and pleural fluid) were identified with new STs. These newly identified STs include ST‐7466, ST‐7467, ST‐7468, ST‐7469, ST‐7470, ST‐7471, ST‐7472, and ST‐7473 (Table 6). New STs that were submitted in Institut Pasteur, Paris, France as following submission (Submission: BIGSdb_20240619114104_3057922_16382, submitter: Hassan Valadbeigi).

**Table 6:** Allelic profile of hvKP isolates found in current study based on Institut Pasteur scheme and newly assigned ST isolates.

	MLST alleles							Isolates	SD
ID	*gapA*	*infB*	*mdh*	*pgi*	*phoE*	*rpoB*	*tonB*	New assigned‐ id	New assigned ‐ SD
10	2	6	70	8	467	81	77	id: 72360.	ST‐7466
14	2	1	469	322	9	1	161	id: 72361.	ST‐7467
17	3	77	3	322	9	4	42	id: 72362.	ST‐7468
25	3	4	435	393	7	48	940	id: 72363.	ST‐7469
43	3	4	435	383	7	6	38	id: 72364.	ST‐7470
46	2	1	2	322	9	1	911	id: 72365.	ST‐7471
58	2	85	435	431	26	4	77	id: 72366.	ST‐7472
60	2	6	38	1	16	7	77	id: 72367.	ST‐7473

**Figure 1:**
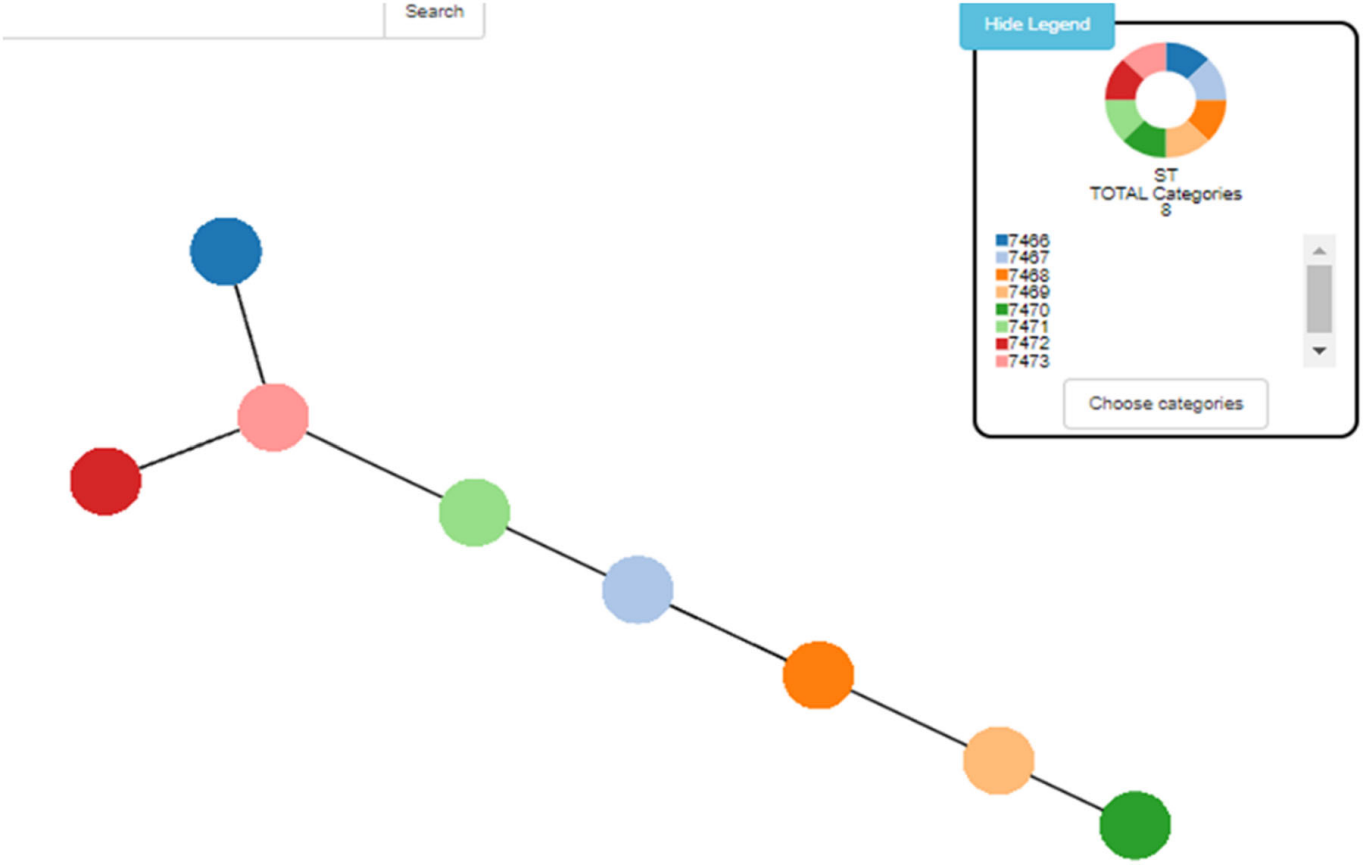
Phylogenetic relationship of new STs of a hvKP by PHYLOViZ software.

We used the PHYLOViZ software (PHYLOViZ Online is freely available at https://online.phyloviz.net) to elucidate the phylogenetic relationship among the new STs (Figure 1).

## 4 Discussion

Hypervirulent *K. pneumonia* has spread worldwide, so that hvKP infections are widely distributed geographically. Therefore, hvKP infections have become a global problem spanning Asia, Europe, Oceania, North America, South America and Africa. The Asia‐Pacific region has the highest prevalence of these infections (Zhu et al. 2021). hvKp is a highly pathogenic microorganism that can cause a range of severe infectious in both outpatients and inpatients, thus become a global public health challenge (Russo et al. 2024). The prevalence of community‐acquired infections caused by hvKP has emerged as a significant clinical problem (Chen and Chen 2021; Li et al. 2014). Rate of prevalence of hvKP infection in china with a varied geographic distribution were reported between 8.33% and 73.9% (Chen and Chen 2021). So, this rapid increase in hvKP infections poses a significant challenge to clinical medicine (Li et al. 2014).

Due to the importance of detecting hvKP, various methods are used to detect hvKP. Both phenotypic methods such as the string test and potassium tellurite resistance and genotypic methods such as PCR can be used for this purpose (Latha et al. 2024). Phenotypic methods are often faster and easier to perform, but they may not be as specific and sensitive as genotypic methods. On the other hand, genotypic methods such as PCR provide more accurate and detailed information on the genetic composition of hvKP (Alharbi et al. 2023; Zhang et al. 2023).

The string test is often used as an initial screening method for the detection of highly pathogenic isolates of *K. pneumoniae (*Latha et al. 2024; Catalán‐Nájera et al. 2017). Fang et al. suggest that the string test is the most appropriate laboratory marker for hvKP *(*Shi et al. 2018 *)*. However, Shi Shi et al. (2018) found that the string test is not a reliable indicator for assessing the virulence level of hvKP. And hypermucoviscosity, which is often associated with hvKP, does not accurately represent hypervirulence and therefore should not be equated with hvKP *(*Shi et al. 2018 *)*. In contrast, our study supports the view of Fang et al. and shows that the string test is a reliable indicator for evaluating hvKP virulence.

MCKT serves as a specialized medium for the isolation and identification of *K. pneumoniae* from environmental matrices as well as from fecal samples from animals and humans (Passet and Brisse 2015). So, MCKT medium was used for detection of resistance to potassium tellurite of hvKP. 46.1% of isolates were resistance to potassium tellurite whereas, accordingly the study by Xiufeng Wu et al. the prevalence of tellurite‐resistant *K. pneumoniae* was 16.7% in fecal specimens from healthy volunteers (Wu et al. 2022).

According to the results of this study, the highest number and percentage and the lowest number and percentage were hospitalized from the samples obtained from urine and tracheal aspiration, respectively. These results show that despite the high percentage of urine samples in this study, the number of in hvKP samples collected from patients was lower than their number. In relation to the tracheal aspiration samples, despite the lower number and percentage of samples, one hvKP sample was also separated. On the other hand, of the 11 samples separated from the plural fluid samples of hospitalized patients, more hvKP samples were separated and with a high percentage (37.5%). Thus, considering that *K. pneumoniae* colonizes the mucous membranes of the human body and that the degree of colonization with this bacterium varies depending on the body site, and that this bacterium is isolated both in the community and in the hospital, it can be related to this. And on the other hand, there are reports of an increase in bacterial colonization in hospitalized patients (Martin and Bachman 2018) and also after antibiotic treatment (Martin and Bachman 2018; Rose and Schreier 1968) and furthermore, bacterial capsule is one of the most important factors that plays a role in the colonization and pathogenicity of this bacterium, therefore, the above reasons may be the reason for the different isolation of more hvKP samples from different sources of the body, as well as the increase in the isolation of samples from different sites of the body, including pleural fluid (Martin and Bachman 2018).

On the other hand, the improvement of methods to detect hvKP could involve the integration of molecular techniques such as PCR to identify virulence genes. The presence of virulence‐associated genes in hvKP provides important insights into its pathogenicity. In contrast to other bacteria that lack these virulence‐associated genes, hvKP exhibits an increased ability to cause severe infection. This includes increased resistance to host immune defenses and a greater ability to invade various tissues. Consequently, hvKP poses a greater threat to human health compared to its less virulent counterparts (Zhu et al. 2021). In this study, the virulence factors *mrkD*, *iroB*, *iucA*, *iutA*, and *rmpA* were examined and were positive results in 11.8%, 7.8%, 23.5%, 9.8%, and 15.7% of *K. pneumoniae* isolates, respectively. These findings emphasize the potential role of these genes in promoting the virulence and pathogenicity of hvKP, such that other study results demonstrate that the genes *iucABCD*, *rmpA*, and *iroB* serve as biomarkers for hvKP (Russo et al. 2018).

MLST provides a highly reproducible and accurate method for characterizing bacterial isolates. In addition, MLST can reveal detailed genetic relationships and track the distribution of specific clones. Based on the results of the MLST, the majority of isolates obtained in this study were found to differ in at least three or four alleles from the closest STs. Consequently, our results led to the assignment of new ST_S_, based on the scheme of the Institut Pasteur, Paris, France. MLST analysis by lee. I.R. et al. revealed a higher ST diversity within the capsule type K2 compared to serotype K1. In particular, ST23 was predominantly associated with serotype K1, while K2 showed associations with several STs, including ST25, ST86, ST375, and ST380 (Lee et al. 2016). The study conducted by Lin et al. also showed that the ST23 strain of *K. pneumoniae* plays an important role in hospital‐acquired invasive infections. This strain was found to have a hypervirulent phenotype (Lin et al. 2018). In November 2020, hvKp ST23 isolates were discovered in Ireland. These hvKp ST23 isolates were found from various sources such as blood cultures, liver abscess, urine, and wound swabs. The isolates were reported as carry hypervirulence‐associated genes: *iroB*, *iroC*, *iroD*, *iroN*, *iutA*, *iucB*, *iucC*, *iucD*, and rmpA2 (ASSESSMENT RR 2024). In previous studies, the genes *iutA* and *rmpA* were identified in 40.9% and 40.4% of *K. pneumoniae* isolates from inpatient samples, respectively (Wu et al. 2022). In the current study, these genes were detected in 9.8% and 15.7% of the isolates, respectively. Thus, the findings of this study differ considerably from the previously reported data.

The main limitation of this study was the small number of samples submitted for sequencing due to lack of funding. Therefore, it is suggested that the large number of samples in the different sources and sites of the clinical unit where the prevalence of *K. pneumoniae* is high be investigated in the future.

## 5 Conclusion

Hypervirulent *K. pneumonia* has become a global public health challenge due to its widespread distribution and high pathogenic potential. The increasing rates of hvKP infections, especially in the Asia‐Pacific region, pose major challenges to clinical medicine. The identification of virulence factors such as *mrkD*, *iroB*, *iucA*, *iutA*, and *rmpA* underscores their critical role in the pathogenicity of hvKP and supports previous findings on the hypervirulent phenotype of certain strains such as ST23. However, the differences in the prevalence of these genes across studies suggest geographic and sample variability. MLST has proven valuable for understanding genetic diversity, with novel STs identified in this study further highlighting the genetic variability of hvKP. Despite these findings, limitations such as small sample size and limited sequencing resources point to the need for larger studies. Future research should focus on broader sampling from high‐prevalence clinical units to better understand hvKP epidemiology and develop targeted strategies for detection and management.

## Author Contributions

**Mohammad Hossein Haddadi:** methodology, data curation. **Nourkhoda Sadeghifard:** validation. **Saeed Khoshnood:** formal analysis, methodology, investigation. **Abbas Maleki:** software, investigation. **Sobhan Ghafourian:** writing – review and editing, formal analysis. **Hassan Valadbeigi:** writing – original draft, writing – review and editing, validation. All authors reviewed the manuscript.

## Ethics Statement

This project was approved by the Ilam University of Medical Sciences Ethics Committee (IR.MEDILAM.REC.1403.090). This study was in accordance with the Declaration of Helsinki.

## Consent

All patients provided written informed consent.

## Conflicts of Interest

The authors declare no conflicts of interest.

## Data Availability

Data sharing not applicable to this article as no datasets were generated or analyzed during the current study.
